# Nursing Partnership Activities, Components, and Outcomes: Health Volunteers Overseas in Uganda 2001–2016

**DOI:** 10.3389/fpubh.2017.00173

**Published:** 2017-07-14

**Authors:** Scovia Nalugo Mbalinda, Rose Chalo Nabirye, Elizabeth Ayebare Ombeva, S. Danielle Brown, Jeanne M. Leffers

**Affiliations:** ^1^Department of Nursing, School of Health Sciences, Makerere University, Kampala, Uganda; ^2^Barrow Neurological Institute at Phoenix Children’s Hospital, Phoenix, AZ, United States; ^3^Department of Community Nursing, University of Massachusetts Dartmouth, Dartmouth, MA, United States

**Keywords:** collaboration, partnerships, education, evaluation, nursing

## Abstract

Nurses increasingly form global health partnerships through academic and voluntary organizations that are designed to improve health outcomes. Many such partnerships are funded for specific time periods and have short- or long-term goals to achieve during the partnership. Other partnerships are sustained for longer periods of time through the efforts of partners committed to their joint work. The case example of the Health Volunteers Overseas Nursing Education partnership in Kampala, Uganda, demonstrates key components of partnerships that promote sustainability of programs. This case example is analyzed using literature that reports partnership models to identify those factors that have led to sustainability. Additionally, both objective and subjective program outcomes are reported. Recommendations for further evaluation are included.

## Introduction

Partnerships are essential to the work of global health professionals to improve health outcomes (1). The organization Health Volunteers Overseas (HVO) works in partnerships comprised of health-care professionals across international borders to strengthen the numbers and capacity of the health-care workforce in resource scarce settings (2). Founded in 1986 with the support of the former Orthopedics Overseas, in 2015, the organization sent 87 volunteers from a variety of health-care specialties to more than 25 countries. Over the past 30 years, this totals more than 10,000 completed assignments (3). HVO is a 501c3 non-profit organization based in the United States (US) that partners globally with host countries at their request. Since the establishment of the HVO Nursing Education Program in Kampala, Uganda, more than 108 nurses have served for stays of 1 month to as long as 6 months. Many volunteers (14) have returned for several volunteer assignments with 1 volunteer serving 12 times for a total of more than 18 months. This paper will examine the key contributions, sustained projects, and outcomes of this collaborative partnership.

## Background

The HVO nursing education program began in Uganda in 2000. At that time, Ms. Speciosa Mbabali, the Acting Head of the Department of Nursing at Makerere University, approached the HVO orthopedics team liaison at Mulago National Referral Hospital to inquire about HVO support for the newly formed undergraduate nursing baccalaureate degree program. Ms. Mbabali recognized that without opportunities in Uganda for higher education in nursing, that a partnership with out of country nurses with advanced degrees was essential in order to build capacity for a strong faculty. HVO sent two experienced nurses, Marie O’Toole, EdD, RN, FAAN, and Julia Plotnick, RN, MPH, FAAN, RADM, USPHS (RET), to collaborate with Ms. Mbabali and the department of nursing at Makerere (4). The continuation of this partnership for 16 years results from some key factors for successful partnerships.

Makerere University is one of the oldest and most prestigious universities in East Africa founded in 1922 in Kampala. Currently, the Department of Nursing is part of the College of Health Sciences with schools of Medicine, Public Health, Biomedical Sciences, and Health Sciences. Dean Rose Chalo Nabirye currently leads the School of Health Sciences, composed of the Departments of Nursing, Dentistry, Pharmacy, and Allied Health Sciences. The Department of Nursing has approximately 12 full-time faculty of whom 3 have now completed a PhD and all others have an MS degree with 4 enrolled in PhD programs.

### Partnership Characteristics

The current literature about partnerships for global health designates key factors that increase the likelihood of successful and sustainable partnerships. Relationship building strategies such as knowledge of the host country social, cultural, economic, and political factors for mutual planning, clear expectations, intentional listening, honesty and openness, mutual trust, cultural respect, and willingness for commitment are integral to partnership formation (1, 5, 6). Continuance of the partnership requires open and 2-way communication, steady leadership, respectful negotiation, teamwork, strong involvement of host partners, program champions, and capacity building in order to build effective collaboration. Commonly reported outcomes of global partnerships are program sustainability, host country ownership, joint publications with shared authorship, ongoing professional development for host partners, strengthened research capacity, and improved curriculum (1, 7, 8).

Since the establishment of the HVO nursing education partnership with the nurse leadership at Mulago National Referral Hospital and Makerere University in 2000, there have been significant projects completed and some that currently continue. The primary goal of the Ugandan partners at the outset was to build capacity for nursing faculty and for clinical nurses at Mulago National Referral Hospital with a focus upon pediatrics due to the high infant and child mortality rates. Data reported in the 2000/2001 UDHS report showed infant mortality rate of 89 deaths per 1,000 live births and under-five mortality rate to be 152 deaths per 1,000 live births. Results reported in the 2011 UDHS report showed a decline in infant mortality of 39% to 54 deaths per 1,000 live births, while the under-five child mortality declined from 152 to a rate of 90 deaths per 1,000 live births. This indicates that about 1 in 18 Ugandan children dies before their first birthday and 1 in 11 Ugandan children dies before their fifth birthday (9).

## Results

The priority for the nursing education partnership in Uganda was to strengthen health-care capacity to improve child health outcomes. Collaborative educational approaches were designed to empower host country nurses for the development of best practices for clinical practice and nursing education. Since that time, the Makerere University faculty-to-faculty partnership projects include (1) improvements to the pediatric nursing curriculum, (2) improvements to the fundamentals of nursing course, (3) introduction of a critical care curriculum, and (4) the establishment of Masters Degree Program. At Mulago National Referral Hospital, the nursing clinical partnerships have been in the following areas: (1) partnerships for the Special Care Babies Unit to build capacity for care of newborns, (2) clinical care for pediatrics, (3) clinical care for critical care, and (4) the diabetes partnership. Using exemplars from four of these collaborative projects, we show how the key factors for successful partnerships have positively impacted the HVO nursing education partnership in Kampala, Uganda. First, we will provide brief descriptions of each project and then highlight the key partnership factors. Finally, we will describe outcomes from the HVO nursing education collaboration (see Table 1).

**Table 1:** Case exemplar partnership and sustainability factors.

Uganda Nursing Education Project	Partnership and relationship building factors	Sustainability factors
Mulago National Referral Hospital Special Care Babies Unit	Cultural respect, trust, open communication, teamwork, and ongoing interdisciplinary collaboration for capacity building and willingness for commitment	Ongoing assessment, open and two-way communication, steady leadership, program champions, teamwork, and strong involvement of host partners
Mulago National Referral Hospital Critical Care Units	Expressed need from Ugandan partners, clear expectations and two-way communication grounded with cultural respect, coordination of activities, and willingness for commitment	Open and two-way communication, program champions, appropriate resources, program champions, and capacity building
Makerere University Pediatric Nursing Curriculum	Needs assessment and joint planning, strong involvement of the Ugandan partners, mutual trust, shared learning, cultural respect, teamwork capacity building, and willingness for commitment	Ongoing assessment, open and two-way communication, program champions, teamwork, strong involvement of host partners, and capacity building
Makerere University Graduate Education Curriculum	Response to an expressed need, mutual trust, shared decision-making, and commitment to the project	Open and two-way communication, appropriate resources, respectful negotiation, teamwork, strong involvement of host partners, program champions, and capacity building

### Mulago National Referral Hospital Clinical Partnership for Special Care Babies Unit

The first project began in 2001 when the HVO nursing education partnership matched two US nurse volunteers with expertise in the care of high-risk newborns, with the Ugandan nurses who worked in the Special Care Babies Unit at Mulago National Referral Hospital. The US nurses joined a US neonatologist on that first as well as many subsequent visits. Mulago National Referral Hospital was founded in 1913 and expanded in 1962. With a capacity of 1790 beds, the Mulago government hospital serves more than 750,000 inpatients and more than 800,000 outpatients annually. Currently, the hospital is undergoing a major renovation at a cost of $40 million. Some clinical services are housed at the “New Mulago” site, the 1962 facility while others are housed across the hillside in buildings that date to 1913. This includes some maternity and most pediatric services as well as clinics and specialty services (10). Both the university and the hospital maintain collaborations with many US universities such as Johns Hopkins, Yale, Baylor, and the University of California San Francisco, as well as the Karolinska Institute in Sweden.

Each of the original nurse volunteers has returned to Mulago regularly since that first visit. One has completed her 12th volunteer assignment in 2016, while the other has returned frequently as well. They additionally bring other neonatal nurse specialists to join their team. The activities and programs that have been built and sustained as a result of this collaboration include formal classes such as newborn resuscitation, clinical education for Ugandan nurses with nurse volunteers working jointly on the unit side by side, and outreach across Uganda for the Saving Babies Lives (11) program. The success of these efforts is due to the long-term relationship built upon cultural respect, trust, open communication, teamwork, and ongoing interdisciplinary collaboration for capacity building. Further, the collaboration has been sustained due to the commitment of HVO and Ugandan nurse champions or leaders. Challenges related to the lack of essential material supplies, inadequate staffing, and lack of other essential resources often threaten the success of the projects. However, this program continues due to the long-term commitment of the HVO volunteers, the long-standing collaboration with both the nurses and the Mulago pediatricians, and the capacity building of the Ugandan nurses working with the high-risk newborns.

### Mulago National Referral Hospital Critical Care Initiatives

Highly trained physicians and nurses in critical care or intensive care units care for patients with life-threatening conditions. These units are well established in high resource settings and are fully equipped with the latest in medical technology. However, these types of units are still in the development phase in Uganda (12). Dedicated physicians and nurses care for patients with systemic trauma, respiratory, and cardiovascular compromise without a sufficient number of ventilators, cardiac monitors, advanced imaging capabilities, and adequate lab support. While generous donors have supplied equipment, the need has been for continuing education to build capacity for the nursing staff caring for these patients.

The collaboration for critical care began in 2013 when a critical care component was added into the undergraduate curriculum at Makerere University. At that time, there were two nurse faculty members with preparation in critical care nursing but they had not yet implemented a critical care curriculum. An HVO volunteer with expertise in critical care nursing came to partner with the Makerere faculty and to jointly design the didactic and clinical experience. The first offering of this clinical component was in March of 2015 to the senior undergraduates. The students completed their clinical rotations in the critical care unit, the pediatric intensive care unit, the dialysis unit, the emergency room, and the trauma unit. The HVO nurse volunteer supported the Makerere nursing faculty and provided direct supervision of the students at the clinical sites at Mulago during the first course offering.

From their observations of the nursing students with the HVO volunteer, the nursing staff in the patient care units at Mulago National Referral Hospital, noting the value of continuing education in the care of the complex, critically injured patient and with the technology being utilized, recognized an available resource. On-site classes were provided by the HVO volunteer in the critical care unit and the trauma unit. This initiative will continue with future nurse volunteers. Nurse to nurse clinical education increases the capacity of the nursing staff in the critical care units.

Future plans include collaboration to develop a Master’s degree in Critical Care Nursing as a second clinical focus of the MS degree in Nursing at Makerere University. HVO volunteers and nursing faculty are in the process of developing the curriculum, collecting the necessary supplies such as mannequins for the simulation lab to teach resuscitation and so forth. This course will complement the support and continuing education for the hospital staff.

The partnership factors that have contributed to the success of this more recent project include the fact that the HVO nurse volunteer responded to a clear need expressed by the Ugandan partners. Further, clear expectations and two-way communication grounded with cultural respect facilitated the coordination of activities at both the university and the hospital clinical sites.

### Pediatric Curriculum Building at Makerere University Department of Nursing

The other early collaborative nursing education project was built from a partnership with an HVO nurse volunteer with expertise in pediatrics who volunteered first in 2002 and continued her collaboration through 2007. During one volunteer assignment of 6 months duration, she was able to partner with the Makerere faculty to develop a comprehensive pediatric nursing curriculum for classroom learning. In 2008, a new volunteer built upon that work extending the curriculum into the clinical setting in collaboration with Makerere pediatric nursing faculty and clinical nurses at Mulago National Referral Hospital. Further activities related to building faculty pediatric expertise included the development of teaching modules for relevant course content, development of faculty supervision of clinical learning at Mulago National Referral Hospital, and a mentorship program using an HVO nurse expert paired with a novice Ugandan nurse.

In this pediatric project, the partnership components included comprehensive needs assessment and joint planning, strong involvement of the Ugandan partners, teamwork, and capacity building that strengthened the collaboration. The needs assessment included collaboration with former US nurse volunteers and communication with Ugandan nurse leaders. Joint planning with the leadership at Makerere University led to extensive preparation of teaching materials for class presentations and handouts, all available on a portable USB drive, edited for appropriateness to the Ugandan health setting to ensure cultural relevance. All materials became part of the Department of Nursing resources to ensure sustainability. Second, meetings with clinical nurse partners at Mulago built trust between the HVO volunteers, the clinical nurse partners, and the pediatric nursing faculty. During three subsequent volunteer assignments over 7 years, the HVO nurse provided guest lectures, clinical supervision, and teaching materials for course updates to sustain the partnership.

### Development of Graduate Education at Makerere University

Many of the Makerere BSN nursing graduates had assumed clinical, leadership, and administrative positions in the Ministry of Health, public and private hospitals, non-governmental organizations, nursing schools, research organizations, and universities. However, despite the outstanding contributions made by the BSN graduates from the Department of Nursing at Makerere University and other universities, health indicators showed an urgent need for nurses trained at the Masters Level for Advanced Clinical Practice Nurses. By 2008, the Department of Nursing had built the capacity of the faculty to include three faculty members who were completing a PhD, and four who had completed an MS degree and had the capacity to begin a graduate program. A goal of that time was to establish a Master of Science in Nursing (MSN) program at Makerere University.

The Department of Nursing administration engaged with its former students, nurses, and stakeholders in the country for a needs assessment. Former and current students and stakeholders raised concerns and urged the department to provide them with an opportunity for a clinically focused Masters Degree in Nursing. In recent interviews with more than one half of the BSN graduates of the Makerere Program, 90% of those who had not acquired a Masters degree stated they were motivated to return to school for a Masters in Nursing. The faculty at MU identified the lack of available clinical specialist at the masters’ level in nursing to serve as educators, clinical practitioners, and nurse researchers as a high priority.

In 2008, an HVO volunteer with experience as a graduate program director and nursing curriculum chair came to work with the department at Makerere. Among her other collaborative assignments, she was asked to write a proposal for an MSN program. Partnership factors that positively affected the collaboration were the response to an expressed need, mutual trust, shared decision-making, and commitment to the project. While this was not the final proposal that led to the MSN program that began in 2011, it helped to provide a template for the faculty to move forward with a partnership to develop an East African joint program where the specialties would be shared across countries to minimize duplication and share resources. Since the Makerere University Department of Nursing had strong capacity for midwifery, that program was launched first.

The Aga Khan University Advanced Nursing Studies Department in East Africa and the University of Nottingham School of Health Science worked on a prestigious European Union funded program called Improving Nursing Education and Practice in East Africa (INEPEA). This ambitious program was supported by the WHO and involved three universities in East Africa, Makerere University, Muhimbili University of Allied Health Sciences, and Zanzibar College of Health Sciences. It resulted in the development of a shared competency framework and MSN curriculum for advanced nursing practice (ANP) in East Africa. The program developed ANP in the East African context. The Master of Nursing-Midwifery and Women’s Health (MN-MWH) was developed to help to address the urgent needs and demands in the country and the region for primary care practitioners who are competent and skilled in midwifery and women’s health. The program also produced nurse educators and scholars, and nurse researchers in Ugandan and regional universities, hospitals, and other settings. The MN-MWH program also provided candidates for doctoral studies in nursing and subsequently researchers to develop locally relevant research in Uganda. The MN-MWH program has prepared students at the postgraduate level and equipped them with clinical, leadership, teaching, and research skills to practice in the primary care settings, in hospitals, schools of nursing, universities, research organizations, and other settings. In addition, the HVO nurse who in 2008 helped develop the first draft of the MN-MWH program proposal returned in 2012 to teach a course for the first cohort of that program.

## Discussion

As an organization of volunteer health professionals primarily from the US and Canada, HVO partnerships during much of this 16-year partnership lacked formal evaluation measurements for each project required of most program partnerships that are funded through public or private sources. Instead, volunteers completed Trip Reports until recently and now complete Volunteer Surveys or for returning volunteers Impact Assessment Surveys in an effort to develop stronger evaluation measurements (13). In addition, HVO requires annual evaluation reports from each Project Director and collectively reports outcomes for each program. The latest Nursing Education Report is from 2015 and notes some of the outcomes discussed in this paper including positive feedback from both the HVO Project Director and the On-site Coordinator (14). Despite the limitations for evaluation of individual projects, many HVO partnerships are able to identify process (or often referred to as formative), impact, or outcome evaluation results. Evaluations for global health partnerships include not only measures to track improved health outcomes, but also elements of sustainability such as building capacity of the host or recipient community, continuation of program activities, host partner ownership of the project, and continuation of program innovations (1, 7, 8, 15).

For most of the duration of the long-term collaboration between HVO nurse volunteers and the Ugandan nurse partners, there has not been a formal evaluation method in place to guide planning and ensure sustainability until recent years. Instead, there have been several structural components that have contributed to continuity. First, HVO nurse volunteers have submitted trip reports that highlight program activities and achievements for each volunteer experience and these are posted on the HVO KnowNet website to allow for future volunteers to build upon the work of prior participants. Second, each HVO program has a US-based Project Director whose job is to coordinate not only the screening of applicants for the program but to work with the host partners to ensure continuance of key collaborative projects or to prepare for new collaborative innovations based upon the skill set of the HVO volunteer. During the 16-year history, two prior Project directors each served for 7 years contributing to the continuity to of the program. The ongoing engagement of the stakeholders in the Department of Nursing with the HVO project directors helps to identify areas of need for support. This continues to involve both groups of partners in planning for future volunteers and projects. Third, HVO sponsors an HVO staff person in Uganda to assist all HVO volunteers coming to Uganda with their professional and logistical accommodations including obtaining a professional license in Uganda, housing, and setting up meetings with professional partners. Each of these processes serves to advance the partnership and collaboration.

### Examples of Outcomes Related to Capacity Building for Ugandan Nurse Partners

Some outcomes of the long-term partnership between HVO nurse education volunteers and the Ugandan nurse partners are objective and could be measured by formal evaluation tools. These include outcome measures such as the number of nurses educated at training sessions, number of courses taught and students in attendance, actual teaching materials and presentations developed for the partnership, number of joint publications, number of joint research projects, and formal feedback from the Ugandan nurses. While some of these objective data are reported in HVO volunteer trip evaluation reports, there has not been a uniform evaluation tool in use across HVO nursing education programs. However, there are sustained outcomes as educational programs continue for each of the projects discussed in this paper (see Table 2).

**Table 2:** Case exemplar outcomes.

Uganda Nursing Education Project	Outcomes
Mulago National Referral Hospital Special Care Babies Unit (since 2001)	Sustained partnership for clinical best practice Newborn resuscitation training Saving Babies Lives training
Mulago National Referral Hospital Critical Care Units (since 2013)	Sustained partnership for clinical best practice Development of critical care course for nursing students In-service education classes for clinical critical care nurses
Makerere University Pediatric Nursing Curriculum (since 2002)	Sustained partnership for pediatric curriculum Development of pediatric course curriculum Development of clinical practice curriculum Formal class lectures Faculty mentorship Host country ownership
Makerere University Graduate Education Curriculum (since 2008)	Sustained partnership from proposal through first cohort instruction Initial Masters Degree Program proposal Ongoing support throughout the proposal process Faculty mentorship for teaching MS courses Host country ownership

Other evaluation data are more subjective in nature. Three significant areas include capacity building for faculty and clinical nurses, engagement and strengthening participation in professional networks, and mutual learning for curriculum design, teaching methodologies, and health-care system approaches. These are particularly challenging to measure as they are highly subjective and internal to both host and volunteer nurse partners. Despite the challenge to evaluate these subjective outcomes, during this 16-year partnership, there are examples of joint publications and support for scholarship. HVO volunteers have served to review manuscripts or conference abstracts for Ugandan partners as well as sharing information about professional meetings of relevance for Ugandan partners.

The Uganda Nursing Education Project Director from 2008 to 2015 sought qualitative feedback from nurses at Makerere University and Mulago National Referral Hospital on each visit to Uganda as well as by email correspondence with the Makerere University Department chair. Two examples of such qualitative feedback are as follows.

Elizabeth Ayebare who was a student and now is pursuing a doctorate wrote:

The Health Volunteer Overseas program is largely responsible for my love of and career as a pediatric nurse. In 2002 when I was an undergraduate nursing student in my third year, I encountered HVO nurse Martha Tanicala, who taught me to love nursing children. Although I loved children from childhood, having a teacher who was passionate about pediatrics was crucial. She nurtured my desire to continue working with children even in my career. I was employed by Makerere University in the nursing department as a teaching assistant/clinical instructor with special focus on pediatric nursing. I used materials developed by Martha Tanicala for teaching and these still act as a reference to date. In 2008 as I figured out my role as a clinical instructor for Pediatric Nursing, I met with HVO nurse Jeanne Leffers who further mentored me to become the clinical instructor I am today. We worked with students together both in class and clinical areas. I had a full time individual mentor with loads of experience and skill. She also provided tools for use, facilitated innovative use of resources in the skills lab and hospital and also helped with assessments. I have since then taken a postgraduate diploma course in child nursing from the University of Cape Town. The HVO volunteers keep coming year after year to give the department of nursing a boost. Currently I am pursuing my PhD on birth asphyxia in the newborn area due to the contribution it makes to under five mortality. I am always grateful to the HVOs for opening up my horizon.

The Dean of the School of Health Sciences, Dr. Rose Chalo Nabirye wrote:

The Department of Nursing at Makerere University has had a long relationship with Health Volunteers Overseas. The volunteers have supported the department in form of human resources when the faculty capacity was limited to effectively teach all the nursing specialties. They helped the faculty with classroom teaching as well as clinical supervision of students especially in medical- surgical, pediatrics and critical care nursing specialties. Working with the HVO volunteers built the capacity of faculty through peer-to-peer learning and mentorship. Further, their vast experience and zeal to work has continued to inspire and develop the passion for nursing among the faculty. When the HVO volunteers observe lack of certain critical supplies for student’s learning, they have often come with them in their subsequent visits. The HVO volunteers have not only supported the department in teaching and student supervision, they have also helped in the development of the Master of Nursing (Midwifery and Women’s Health) curriculum (2008-2011). This program was the first clinical Masters in Nursing at Makerere University, Uganda in 2011. The HVOs were also involved in the operationalization of the curriculum for the first cohort and continue to participate in the teaching of this program.

One important feature of the partnership has been the ongoing relationship between several nurse volunteers and Ugandan nurse faculty members. This has been aided not only by the return visits to Uganda by many of the volunteers over 3- to 15-year time periods but also with the aid of technology such as email, Skype, and cell phones to sustain the partnership over time (4).

Further, the partnerships impact the HVO nurse volunteers in a variety of ways that are difficult to measure. First, in their trip report evaluations, they noted that the opportunity to live and work in a culture very different from their own culture and work environments expanded their understanding of cultural practices and health-care systems. Second, they learned to become more adaptable in planning nursing care due to the lack of material resources and technology they had been accustomed to at home. Finally, for some, the partnership fostered opportunities for research and scholarship.

Outcome measurement of the HVO/Uganda partnership in Kampala is complicated by the fact that the contributions have not been made in isolation. During the same time period that HVO nurse volunteers offered support to the Makerere University nursing faculty and Mulago National Referral Hospital clinical nurses, many other partnerships were operating as direct collaborations between academic or clinical partners throughout Africa as well. The capacity built by intra-continental collaboration as well as global partnerships all were influential in strengthening capacity. The long-term partnership with the Karolinska Institute of Sweden included bilateral exchange for both nursing faculty and students alike as well as opportunities for support for higher education, research, and scholarship. The Bill and Melinda Gates Foundation “Partnership for Building the Capacity of Makerere University to Improve Health Outcomes in Uganda,” a grant funded Collaborative Learning Initiative with Johns Hopkins University (14) provided both human and material resources over the 3-year grant cycle. Other opportunities for higher education, research partnerships, and program initiatives with a variety of eastern and southern African institutions also advanced the capacity of Ugandan nurses.

### Recommendations and Future Implications

The examination of this case example of the partnership between HVO nurse volunteers and the academic and clinical nurses at Makerere University and Mulago National Referral Hospital in Uganda offers global health professionals lessons for future partnerships and program sustainability. Capacity building remains a high priority to build stronger academic programs (16). Spies et al. (17) report that clinical practice and nursing education are priority areas for nursing research and that research findings must be translated into practice and policy development to advance the profession and to improve health outcomes (17). One recommendation for future nurse volunteers would be to work with the Ugandan nurses for collaborative research and scholarship. Ugandan nurses and health professionals report that research priorities and research utilization in clinical practice are high priorities for future work (16, 17).

A second recommendation is that formal evaluation tools be developed by HVO for use with specific projects. These would support the evaluation measures currently in place (13, 14). This could include quantitative measures such as the numbers of educational offerings, attendees, curricular innovations, joint research projects, joint publications as well as post presentation surveys. In addition, subjective measures such as open-ended questionnaires, exit interviews, and other feedback would help demonstrate partnership effectiveness.

Finally, a pre-trip preparation tool to supplement the resources available on the HVO KnowNet link on the HVO website would be helpful for new volunteers in order to learn about ongoing projects and allow them to increase their effective contributions both while in country and as ongoing partners. The tool could include content areas for the new volunteer to complete related to history and culture of the host setting, health-care system information, and summary of host country ongoing projects with a focus upon components for successful partnerships in order for the volunteer to prepare fully for the assignment. Both the pre-trip and post-trip evaluation tools could be useful for all HVO projects across countries and professional disciplines.

## Conclusion

The case example of the nursing partnership between HVO volunteers and Ugandan nurses in Kampala, Uganda, advances knowledge of partnership factors that promote successful partnership and sustainability. Although outcome measurement is more difficult than for shorter term funded projects with defined goals and outcomes, the ability to sustain the partnership over almost two decades fosters greater potential for new innovations over time. Long-standing relationships between health professionals from visiting and host settings promotes the honesty and openness, clear communication, mutual trust, cultural respect, and strong involvement of host partners necessary for relationship building to create successful partnerships.

## Author Contributions

SM: author, manuscript writing, revision of the manuscript, and final approval of the submission. RN: co-author, manuscript writing, revision of the manuscript, and final approval of the submission. EO: co-author, manuscript writing, and revision of the manuscript. SB: co-author, manuscript writing, revision of the manuscript, and final approval of the submission. JL: senior author, manuscript writing, critical revision of the manuscript, and final approval of the submission.

## Conflict of Interest Statement

The authors declare no commercial or financial relationships related to the program reported in this manuscript that could be construed as a potential conflict of interest.
